# Editorial: Insulin Resistance, Metabolic Syndrome, and Cardiovascular Disease

**DOI:** 10.3389/fcvm.2022.959680

**Published:** 2022-06-22

**Authors:** Tong Yan, Yuli Huang, Jason H. Y. Wu, Xiao-Dong Zhuang, Xiong-Fei Pan

**Affiliations:** ^1^Department of General Surgery, Center for Obesity and Metabolic Health, The Third People's Hospital of Chengdu and the Affiliated Hospital of Southwest Jiaotong University, Chengdu, China; ^2^Department of Cardiology, Shunde Hospital, Southern Medical University, Guangzhou, China; ^3^The George Institute for Global Health, Faculty of Medicine, University of New South Wales, Sydney, NSW, Australia; ^4^Cardiology Department, The First Affiliated Hospital of Sun Yat-Sen University, Guangzhou, China; ^5^Key Laboratory of Birth Defects and Related Diseases of Women and Children (Sichuan University), Ministry of Education, West China Second University Hospital, Sichuan University, Chengdu, China; ^6^Shuangliu Institute of Women's and Children's Health, Shuangliu Maternal and Child Health Hospital, Chengdu, China

**Keywords:** insulin resistance, metabolic syndrome, cardiovascular disease, treatment, risk factors, outcomes

Two-hundred and eighty-six authors contributed 35 articles to the Research Topic, Insulin Resistance, Metabolic Syndrome, and Cardiovascular Disease, which received 70,189 views as of April 20 2022.

Heart Failure (HF) could be caused by multiple risk factors, and its treatments are still evolving. Six articles in this Research Topic studied the progression and outcomes of HF. Controversial evidence from prior observational studies indicated that the components of metabolic syndrome (MetS), including hypertension, obesity, and hypercholesterolemia, might have a protective effect on the prognosis in patients with established HF (“epidemiologic paradox”). Huang Z.-M. et al. found that MetS was not associated with all-cause or cardiovascular mortality in HF patients, but was with an increased risk of composite cardiovascular events. Findings from the meta-analysis therefore challenged the “epidemiologic paradox” in the relationship between HF and MetS. In the National Health and Nutrition Examination Survey (NHANES) 2007–2016, patients with chronic HF exhibited higher body mass index (BMI) with lower energy and macronutrient intake, lower physical activity level, and longer rest time and hemodilution (Xu et al.). In addition, the HF patients with BMI ≥ 30 kg/m^2^ had higher water intake, sedentary time, and hematocrit, while intakes of energy and macronutrient, and physical levels were comparable with patients with BMI <30 kg/m^2^. In a review article, Zeng Q. et al. assessed the mechanisms and perspectives of sodium-glucose cotransporter 2 (SGLT2) inhibitors, a novel class of oral antidiabetic medication, in patients with HF. Multiple clinical trials have shown a pronounced decrease in hospitalization due to HF shortly after starting use of SGLT2 inhibitors. SGLT2 inhibitors may exert their benefits in HF by targeting inflammation, oxidative stress, insulin resistance (IR), energy metabolism, advanced glycation end products, obesity, and hyperuricemia. In a retrospective case-control study of 642 patients with rheumatic heart disease (RHD) and HF, Liu C. et al. reported that continuous use of digoxin was significantly associated with increased all-cause mortality, cardiovascular disease (CVD), medium-/long-term HF re-hospitalization, and new-onset atrial fibrillation (AF) in RHD patients with HF, compared with the intermittent digoxin regimen. In a study among 277 patients with HF at various stages, Cao et al. showed that serum secreted frizzled-related protein 2, which may play an important role in the pathological mechanisms of MetS and CVD, was a risk factor for HF in patients with CVD, especially with type 2 diabetes. One novel article based on experiments using *Seipin* knockout mice reported that *Seipin* deficiency might promote the pathogenesis of cardiac hypertrophy and diastolic HF by inducing myocardial calcium handling impairment, endoplasmic stress, inflammation, and apoptosis (Wu X. et al.).

Acute myocardial infarction (AMI) and acute coronary syndrome (ACS) are major causes of death and disability and the primary cause of death in patients with type 2 diabetes. Five articles in the Research Topic addressed this condition. Cui et al. reviewed the latest developments in the research on type 2 diabetes and myocardial infarction (MI), including epidemiology, clinical features, and cardiorenal benefits of the newer class of hypoglycemic agents. Recent evidence of cardiovascular safety and benefits of hypoglycemic agents, including SGLT2 inhibitors, dipeptidyl peptidase-4 inhibitors, and glucagon-like peptide 1 agonists, was discussed in the review. In another review article, Li, Chen, et al. examined stress-induced hyperglycemia (SIH) in the setting of ACS. The article began with various methods used to define SIH in the context of ACS and their pros and cons in its definition, such as blood glucose on admission, glycemic GAP/SHR, and glucose variability. In addition, they summarized major clinical trials involving participants with both AMI and SIH that evaluated the effects of insulin-based therapy on outcomes of ACS, including the DIGAMI, HI-5 study, and BIOMArCS-2 trial. In terms of the mechanisms underlying the harmful consequences of SIH, oxidative stress could play an important role in myocardial reperfusion injury and remodeling. While diabetes is a risk factor for CVD, Zhang et al. reported that severe stress hyperglycemia (≥200 mg/dL or 11.1 mmol/L) in patients without diabetes enrolled in a coronary care unit had an increased risk of the short-term death compared with patients with diabetes. Meanwhile, normal blood glucose and moderate stress hyperglycemia (140 mg/dL ≤ random blood glucose ≤ 200 mg/dL, or 7.8 mmol/L ≤ random blood glucose < 11.1 mmol/L) had no adverse impacts on short-term outcomes in the patients. In a retrospective study (Li, Gao, et al.), new-onset AF occurred more frequently when complicated with elevated fasting glucose (≥7 mmol/L) in patients with AMI, and every 1 mmol/L increase in fasting glucose was associated with a 5% higher rate of new-onset AF. Furthermore, patients with AMI complicated with elevated fasting hyperglycemia and AF had a worse prognosis in terms of in-hospital and long-term all-cause mortality. In addition to hyperglycemia, an article by Zheng S. et al. reported the impact of systolic blood pressure (SBP) on the prognosis of AMI. SBP showed a non-linear effect on AMI prognosis. Compared with normal SBP (90–140 mmHg), both low (<90 mmHg) and high (>140 mmHg) SBP had a worse prognosis in AMI patients, especially in those with a low SBP.

Three articles in the Research Topic were on percutaneous coronary intervention (PCI). Recent studies suggested that lipoprotein(a) and lipid parameters other than low-density lipoprotein cholesterol (LDL-C) could predict CVD, even in patients receiving statin therapy (1–3). However, in a retrospective study among 445 patients with a median follow-up of 36 months, Zeng, Ye, et al.  found that baseline LDL-C showed a better predictive ability for major adverse cardiovascular events in statin-naïve patients after PCI than other non-LDL-C lipid parameters in Chinese. Hence, LDL-C remains a strong predictive factor for CVD events in CVD secondary prevention. Zhao et al. conducted a retrospective study to compare the differences in the long-term prognosis of diabetes and non-diabetes patients who underwent PCI for *de novo* lesions and late or very late stent thrombosis. Diabetes patients with *de novo* lesions showed a higher risk of composite clinical outcomes than their non-diabetes counterparts, and patients with late or very late stent thrombosis lesions had higher risks of long-term composite clinical outcome events compared with patients with *de novo* lesions. Finally, Li et al. introduced a study protocol for a retrospective, multicenter analysis to develop and validate the risk prediction model for new-onset diabetes after PCI.

A meta-analysis by Li X. et al. assessed the impact of MetS and its components on the prognosis in patients with CVD. Fifty-five studies with 162,450 patients from 25 countries and regions were included. MetS was associated with increased risks of all-cause mortality, CVD mortality, MI, stroke, target vessel revascularization, and HF in CVD patients. Regarding the impact of individual MetS components on CVD, higher BMI (>25 kg/m^2^) was associated with lower all-cause mortality, which could be potentially caused by reserve causality, while dyslipidemia and abnormal glucose metabolism were the main risk factors for CVD outcomes.

Many metabolic conditions could worsen vascular lesions. Zheng H. et al. used clinical data of patients with acute thoracic aortic dissection (AD), an animal model of IR and AD, and an IR model of human aortic vascular smooth muscle cells (VSMCs) to explore the role of IR in the pathogenesis of AD. The authors reported that IR could induce the phenotypic switching of VSMCs from contractile to synthetic, which contributed to the occurrence of AD. It was well accepted that metabolic abnormalities could contribute to thoracic aorta calcification (TAC). Liang et al. reported that patients with metabolic abnormalities had higher TAC scores than those without metabolic abnormalities, and β-hydroxybutyric (BHB) was a protective factor for TAC after adjusting for potential metabolic parameters. Using calcification models with arterial rings and VSMCs, the study showed that BHB might serve as an effective intervention target for high phosphate-induced vascular calcification *via* regulating VSMC phenotypical transition. In addition, the enhancement of autophagy in VSMCs by BHB could be the mechanism contributing to the inhibition of arterial calcification.

Endothelial progenitor cells (EPCs) are involved in the regeneration and repairment of adult vessels, and inflammation could potentially impair the function of EPCs. In a clinical study of 34 patients with acute AD and 20 non-AD patients with essential hypertension, Huang Z. et al. reported that the EPC number, migration, and proliferation were significantly reduced in patients with acute AD, and inversely correlated with the AD detection risk score. In addition, the IL-6 and IL-17 levels were negatively correlated with circulating EPC number and migration or proliferation in acute AD, indicating a possible inflammatory mechanism in circulating EPCs in the acute AD. In addition, Zeng, Zhang, et al. showed that the age-related decrement in the EPC function was related to the severity of non-ST elevation MI in men patients, which might also be due to systemic inflammation.

In a prospective population-based study of 7,104 Chinese individuals, Shi et al. reported that the level of proprotein convertase subtilisin/kexin type 9, a key LDL-C mediator, was positively associated with worse cardiometabolic risk profiles in women but not men, including high LDL-C, elevated triglycerides, hypertension, type 2 diabetes, and MetS.

Ectopic fat depots are associated with an increased cardiometabolic risk, and the accumulation of epicardial adipose tissue (EAT) underlies cardiac dysfunction in the population with obesity. Maimaituxun et al. studied the impact of epicardial adipose tissue volume (EATV) on the cardiac function. In this retrospective study among 180 patients with a mean BMI of 25 ± 5 kg/m^2^, the authors found that EATV was independently associated with global longitudinal strain despite preserved left ventricular ejection fraction and a lack of obstructive coronary artery disease (CAD), which suggested that EAT might affect myocardial systolic function through impaired left ventricular longitudinal strain.

CVD and microvascular complications are leading causes of morbidity and mortality for individuals with type 2 diabetes, and four basic and one clinical studies addressed on diabetic complications. The hallmark of diabetic cardiomyopathy is calcium mishandling in cardiomyocytes. Deng et al. showed that calcium dobesilate (CaD), a drug commonly used to treat diabetic microvascular complications, could attenuate high glucose and high lipid-induced impairment in calcium signaling and reactive oxygen species (ROS) production in cardiomyocytes. Thus, CaD might offer a protective effect in diabetic cardiomyopathy. Yan et al. demonstrated that metformin activated the AMP-activated protein kinase (AMPK)—phosphorylating PDZ and LIM domain 5 (Pdlim5) pathway, which could interrupt the migratory machine of VSMCs and inhibit cell migration *in vitro* and *in vivo*. Metformin seemed to suppress diabetes-accelerated atherosclerosis by the maintenance of AMPK activity. In another study on diabetic cardiomyopathy, Liu M. et al. showed that Adropin, a regulatory polypeptide involved in energy hemostasis, lipid metabolism, and IR, could alleviate myocardial fibrosis and improve diastolic function in the diabetic cardiomyopathy rats. Due to the substantial consequences of CVD in patients with type 2 diabetes, early detection of subclinical myocardial changes has important clinical implications. Li W. et al. reported that the combination of real-time three-dimensional echocardiography and myocardial contrast echocardiography in the left ventricle could detect subclinical myocardial dysfunction and impaired myocardial microvascular perfusion. Another article on diabetic complications assessed the mechanism of diabetic retinopathy. Li H. et al. showed that CCN1, which is involved in angiogenesis and fibrosis of retina, activated the NOX4/ROS axis in microvascular endothelial cells and led to vascular leakage through inhibiting VE-cadherin expression. Hence, the up-regulation of CCN1 expression could contribute to the pathogenesis of diabetic retinopathy.

Adequate glycemic control is the cornerstone in the management of diabetes. Numerous clinical trials demonstrated that controlling the blood glucose within the target range would help prevent or attenuate diabetic complications. The most commonly used parameter for assessing glycemic control is the glycated hemoglobin A1c (HbA1c) measurement. Liu L. et al. reported a U-shaped relationship between HbA1c and long-term all-cause mortality among 37,596 patients with CAD during a median follow-up of 4 years. Both low (< 5.7%) and high (> 6.7%) HbA1c levels were associated with increased all-cause mortality compared with the reference group (5.7 < HbA1c ≤ 6.1%). Besides HbA1c measurements alone, Huang D. et al. found that long-term visit-to-visit HbA1c variability was also associated with higher incidence of cardiovascular complications and all-cause mortality in type 2 diabetes in a *post-hoc* analysis of the ACCORD population. Over a median follow-up of 4.85 years, the hazard ratios for the primary outcome (the first occurrence of non-fatal MI, non-fatal stroke, or cardiovascular death) in the second, third, and the highest quartiles of the variability of HbA1c were 1.26 (95% CI 1.03–1.54), 1.24 (95% CI 1.01–1.52), 1.61 (95% CI 1.29–2.00). Although HbA1c is an index that reflects the mean blood glucose for the prior 2–3 months and the gold parameter for evaluating overall glucose control, mean blood glucose could only contribute to 60–80% of the variance in HbA1c. Hempe et al. (4) introduced the hemoglobin glycation index (HGI), a biomarker of population variation in HbA1c due to factors other than blood glucose concentration. Wu J.-d. et al. conducted a meta-analysis to examine the association between HGI and risks of CVD and all-cause mortality in 22,035 patients with type 2 diabetes during a median follow-up of 5.0 years. After adjusting for multiple traditional cardiovascular risk factors, the level of HGI was positively associated with risks of composite CVD and all-cause mortality, and the associations became non-significant when HbA1c was further adjusted for. Thus, HbA1c might drive the association between HGI and CVD in type 2 diabetes.

Five articles reported associations of triglyceride glucose (TyG) index, a novel surrogate marker of IR, with various cardiometabolic conditions. Chen et al. assessed the association between TyG and risk of new-onset diabetes among Chinese adults. Using data from the China Health and Retirement Longitudinal Study, the authors found that a higher TyG index was associated with an elevated risk of new-onset diabetes in middle-aged and older Chinese adults. The effect of the TyG index on diabetes was not modified by age, sex, BMI, glycemic status, fasting blood glucose, or triglyceride level. Liu X.-c. et al. using data from the NHANES 1999–2014 demonstrated non-linear relationships of the TyG index with all-cause mortality and mortality from CVD among the US general population. In an analysis of data from 409 patients who underwent septal myectomy due to hypertrophic obstructive cardiomyopathy, Wei et al. reported that postoperative atrial fibrillation occurred more frequently in the high TyG index group. In the receiver operating characteristic curve analysis, the TyG index could provide a moderate predictive value for postoperative atrial fibrillation after septal myectomy. In a retrospective cohort study of 103 patients with HF who had received cardiovascular magnetic resonance (CMR) imaging examination, Yang et al. concluded that the TyG index was positively associated with myocardial fibrosis detected by CMR. A higher TyG index was also associated with poor prognosis in HF patients after adjusting for other risk factors, including diabetes. In a meta-analysis of 12 studies involving 28,795 patients with CAD, Luo et al. showed that the elevated TyG index was associated with an increased risk of major adverse cardiovascular events, MI, revascularization, and stroke in patients with CAD.

Li et al. a systematically reviewed the relationships of oral, tongue-coating microbiota with metabolic disorders. First, oral microbiota disorders have been reported to be related to diabetes, obesity, CVD, cancer, and other systemic diseases. Second, the mechanism by which the tongue coating microbiota affects metabolisms may be similar to intestinal microbiota, including but not limited to the biological barrier effect, involvement in the nitric oxide cycle, and taste production.

In conclusion, this Research Topic contains a wide range of articles that span from original research to reviews, from animal to human studies, from *in vitro* to *in vivo* experiments, and from small clinical studies to large population-based studies. Evidence in this collection makes a significant contribution to our understanding of the relations among IR, MetS, and CVD.

## Author Contributions

All authors listed have made a substantial, direct, and intellectual contribution to the work and approved it for publication.

## Conflict of Interest

The authors declare that the research was conducted in the absence of any commercial or financial relationships that could be construed as a potential conflict of interest.

## Publisher's Note

All claims expressed in this article are solely those of the authors and do not necessarily represent those of their affiliated organizations, or those of the publisher, the editors and the reviewers. Any product that may be evaluated in this article, or claim that may be made by its manufacturer, is not guaranteed or endorsed by the publisher.
